# Bibliographical Notices

**Published:** 1837-10-01

**Authors:** 


### Periscope;

OR,

### CIRCUMSPECTIVE REVIEW.

"Ore trahit quodcunque potest, atque addit acervo."

### Notices of some New Works.

### THE CYCLOPÆDIÆ.

I. The Cyclopædia of Practical Surgery, comprising a Series of original Dissertations on Operative Medicine, by an Association of Physicians and Surgeons. Edited by *William B. Costello*, M.D. &c. &c. Part II. August 1837.

The second part of this Cyclopædia is now before the public. It contains the following articles by the following authors—Amenorrhœa, by Dr. Jewel; Ammonia, Ammoniac, Ampulla, by Dr. Costello; Amputation, by Dr. King and Dr. Costello; Amylum and Anasarca, by Dr. Costello; Anastomosis, by Dr. Wardrop; Morbid Anatomy, by Dr. Hodgkin; Surgical Anatomy, by Dr. Tarral; Aneurysm, by Dr. Wardrop.

The longest and most important article is that on amputation. Those on morbid and on surgical anatomy are mere skeletons, or rather indices of what the nature of such subjects is. The article on aneurysm is only commenced.

The remarks on amenorrhœa, by Dr. Jewel, are brief, but judicious, perhaps not the less so because they are brief. Dr. Jewel very properly insists on the necessity of looking to the general health in investigating the causes, and in prosecuting the treatment of amenorrhœa, and in the propriety, we may say necessity, of ascertaining the condition of the parts, when the usual remedies fail. His directions with regard to treatment are sensible and practical.

The article Amputation is got up with much care, and illustrated with numerous wood-cuts. These are highly valuable adjuncts, and the publishers will do well to display liberality in the supply of them.

The plan pursued is as follows. The subject is introduced with some general remarks on the limbs; then we have a historical notice of amputation; then the cases in which amputation ought to be performed; then the prognosis of the operation; then the time and the place for amputating; then the preparation of the patient, and the necessary apparatus; then the means of arresting hæmorrhage; then the general methods of amputating; then the dressings after amputation; then the consequences, good and bad, of the operation; then the particular amputations. Supposing such a mode of treating the subject to be copious and accurate in detail, it must obviously be a very complete one.

An analysis of papers of this description is impracticable—a formal criticism tiresome, if not unnecessary. All that can be done is to select a few passages for the purpose of exhibiting the style in which the subject is discussed, and to pick out any novelties of consequence for extract.

*Cautions against unnecessary Operations.*—At the present time of day such remarks as the succeeding are not without value, and may not be without effect :—

" In cases of doubt, the surgeon will, therefore, abstain from using the knife. As we have dwelt a good deal upon the severity of the loss of a limb, with a view to impress the practitioner with the nature of the weighty duty he has to perform, when called upon to decide whether amputation is proper or not; and in order that he may give the subject his utmost attention, we are bound also to remark, that he must not allow his judgment to be influenced by considerations arising from a mistaken notion of what humanity dictates, and which are sometimes pressed by others. What is right is humane, in this as in most other cases. It is humane to amputate, and inhuman not to imputate where the necessity is pointed out by the rule we have supported, just as much as it would be inhuman to amputate, and humane not to amputate whenever the rule does not prescribe the operation. It is not in treating of this operation that we need defend the profession against the imputation, sometimes attempted to be cast upon it, of allowing the fondness for operating to influence its decisions. The amputation of a limb is always a painful duty to the surgeon. In some operations it is not so: in removing a tumour dangerous to life, he may, indeed, execute the task with a feeling of satisfaction; for he knows that he is extirpating morbid matter, that he is dislodging an enemy. But when amputating a limb, he feels that he is taking away an integral part of the body, which cannot be replaced, for the uses of which there is no substitute, and for the loss of which there can be no compensation."

It is most true that, on the part of the profession generally, there is any thing but a fondness for operations ; but it is not so certain that individuals are free from so serious a fault. The reports in the journals contain too many instances of operations performed for the sake of display, or at all events of operations performed under circumstances most unfavourable to the patient. This is a state of things which on all accounts should not be tolerated ; it is disgraceful to the parties, injurious to the profession, and shocking to humanity. It will be put down. Public opinion is too strong for any man to fight against it ; and, when sanctified by the principles which our feelings and our judgment both pronounce to be right, it will vanquish both the artifices and the clamour of faction.

*Amputation during Suppuration.*—" In the few instances of extensive suppuration, or ulceration, for which amputation is justifiable, the time must vary according to the circumstances of each particular case. M. Blandin endeavours to establish a division of suppuration into acute and chronic.—The terms are not strictly appropriate ; because all the distinction that can be admitted is that between suppuration of long standing, and suppuration from a recent lesion. He would call chronic that suppuration which results from scrofulous inflammation in the lymphatic glands, and acute that which occurs to separate the eschar of an extensive burn. The period for amputating in cases of extensive suppuration of long standing is correctly stated by M. Blandin and others, to be when the patient is somewhat, but not too much weakened by the purulent secretion : in cases of recent suppuration, on the contrary, the operation should be performed as soon as it is ascertained to be necessary. For those cases requiring amputation, in which the surgeon has some choice as to the precise period of performing it, and is not strictly tied to time, he should follow the rules laid down for the performance of operations generally : these he ought the more strictly to adhere to, as among what may be termed the major operations of surgery, there is not one of greater consequence than amputation.

He must not forget that a mild temperature and settled weather are decidedly favourable to success. He ought also to bear in mind that it is better to operate in the morning than at any other part of the day. The morning is the more necessary, as the immediate preparations for the operation require some time; and the accidents which may supervene after it (which often require considerable time also) are much better counteracted during the day than in the evening."

The question of the superiority of amputations in the continuity of a limb, or in the joints, is decided by the authors, we think very justly, in favour of the former. But as most English surgeons have been for some time of this way of thinking, it is not necessary for us to go into the arguments in favour of it.

*Preparation of a Patient previously to Amputation.*—This is of great consequence, of very great consequence. Let us hear what Messrs. King and Costello have to say:—

"The body should be prepared by the best measures time and the circumstances of the case admit of; they should be such as are most likely to establish and preserve a good and calm state of the principal functions. In general the quantity of food should be diminished a good deal, but not changed to the extent recommended by the French, who go too far in this respect, while the English perhaps do not go far enough. There is a just medium to be observed. More is certainly to be feared from too much fullness, improperly called strength, than from too much emptiness. The digestive organs should have something to do, but their work ought to be light: the food should be simple, easy of digestion, and of a nature to procure a motion once in twenty-four hours. The circulating system should not be too full; but as some blood must be lost during the operation, and as the quantity can be increased by allowing the small vessels to bleed for a certain time before the ligatures are applied, preparatory blood-letting is seldom or never necessary. Besides, it might not be always safe, as the precise quantity of blood lost during the operation cannot always be foreseen or regulated, although we believe surgeons might regulate it better than they do, if they attended more to this matter, which, at present, they neglect to an unjustifiable degree.

As to the more immediate preparations, it is requisite to see that the bowels act within at least eight or ten hours of the time for operating. Should they not act naturally, it will be proper to procure a motion by an enema. We do not think the habit of purging patients, prior to great operations, a good one: surely it cannot be right to throw important organs into a state of commotion at such a time. No food should be put upon the stomach within three or four hours of the time fixed for the operation."

It, at first sight, appears probable that, as inflammatory sequelæ are what we most dread from amputation, the tendency to them would be diminished by lowering treatment prior to its performance. Yet we are sure that, in practice, such anticipations are not realized, and that more mischief is done by putting the patient in too low than in too high a state. In large towns especially this is found to be the case; and moderately good regimen both before an operation, and as soon as possible after it, conduces to the occurrence of what is so desirable —union by the first intention. We are confident that much of the notorious ill success which follows the great operations abroad, results from their mischievous interference with the natural powers. They always dread too much inflammation, and they take the means of ensuring the least desirable form of it —the suppurative.

The object of purging a short time prior to great operations, is, to prevent the necessity of purging soon after them. Surely the former is in every respect preferable. If there is to be a "commotion," it were best avoided when the cut limb and the dressings may be disturbed by it.

*Hints with respect to the Apparatus.* After observing that the requisite apparatus should be methodically laid out previous to the operation, the authors go on to remark:—To be the more certain that all the instruments are there, the assistant should not neglect to recapitulate the divers steps of the operation, taking up each instrument in the order in which it is required and putting it down again; the operator ought to do the same immediately before he begins the amputation, in order to guard against any omission, which might be of serious consequence. Dupuytren never neglected these precautions.

The best amputating saw, our authors rightly state, is that invented by Dr. Fox of Derby. Its back is joined to the blade only at the extremities, and being moveable, may be inclined on either side so as to admit of the instrument being worked at the bottom of a hollow wound without the least chafing on the muscles or retractor. By this most useful improvement, Dr. Fox has rendered an important service to surgery. The next best saw to that of Dr. Fox is a common sash-saw rather widely set. Indeed, the surgeon should never use one that is not set sufficiently to work freely, or one that he is not accustomed to use. Every saw is an instrument with which a particular acquaintance is desirable on the part of the person who makes use of it. A joiner cannot work well if he has not his own favourite saw: how then can a surgeon expect to do so? A little farther on, our authors dwell on the fact so frequently observed—that a surgeon seldom saws well. The reason why a joiner does so, is, because he learns to saw—the reason why a surgeon bungles, is, because he does not learn to saw. Mr. Guthrie's advice is very good. He recommends surgeons to buy mopsticks, or to steal them, and to saw them up until they can saw them well.

*Compression of the Artery.* The following observations are judicious.

" Just at the instant the incision is about to be commenced, he is to compress the vessel to this degree; and in answer to the interrogatory look or word of the operator, to intimate that he has the circulation in the limb at his command. The pressure should be equal, and made perpendicularly to the plane on which the artery rests, but rather from than towards any accompanying vein. The hand exercising it should be in the most easy position, and so placed that the ends of the finger fall plump upon the vessel.

It will tire too soon if held in a cramped or uneasy position, or if too much force be employed. When the assistant feels fatigue, he can change hands without letting the artery escape, by sliding one down close upon the other. With these precautions, pressure may be kept up for hours.* An experienced assistant can exercise pressure for a very long time without fatigue, because he uses only the necessary degree of force, and takes care to hold himself and his hand in an easy posture. This is the plan of preventing hæmorrhage during amputation which is usually employed in the French hospitals, and which some English surgeons adopt.

Those who have never seen it tried will be agreeably surprised to find with what ease and certainty the passage of the blood may be completely stopped in a large artery by the fingers properly applied, provided the vessel rest upon something solid, as the femoral artery does on the os pubis, and the brachial artery on the upper third of the humerus. The great requisites are, to place the fingers directly over it, as when we feel the pulse at the wrist, to make the pressure perpendicularly to the resting-place, and to use a regular, and only the necessary degree of force."

" The tourniquet ought not to be applied until the surgeon is ready to begin his incisions: he should make it the last thing, so that the moment he has ascertained the instrument to be well fixed, and has confided it to the care of the

---

* " In a case where it was necessary to stop the passage of the blood in the femoral artery, for several days without impeding its venous circulation or injuring the skin, Dupuytren employed several assistants, who effected the object in this way. They relieved each other in turns every three or four hours."

assistant, he may begin the operation. To cause the blood to stagnate in the limb, as some surgeons do, by applying the tourniquet before they are ready to operate, can be of no use, but, on the contrary, must be injurious."

*Vicious Amputations of Dupuytren.* Mr. King is the speaker.

"His incisions were made with such velocity, that the eye could scarcely follow them; and to superficial observers, to those who were mere spectators, his amputations appeared splendid. The dexterity of the operator excited their admiration. But in myself, who assisted Dupuytren in these operations, the admiration was mingled with regret. I had to watch and dress the patients, and I could not shut my eyes to the evil results. The stumps were often conical in the wrong sense; sometimes even the bone was salient and afterwards exfoliated. Those who witnessed the operation only, went away with the notion that Dupuytren's amputations were equal to his other operations, almost all of which were excellent. This was unfortunate; for had it been otherwise, the general opinion of the profession would have been against his plan, and he might have been induced to alter it. His great fault lay in not devoting his first incision entirely to the division and preservation of the integuments. This he either did not see, or thought he could counteract by preserving an additional quantity of muscle; for he was aware that his stumps were often faulty; and I have frequently seen him make more than three sections of the muscles, and also dissect up round the bone, whispering as he proceeded, 'Surely I shall preserve soft parts enough here!' as if determined to secure a good stump."

These are all the observations we think it necessary to extract from this article. It is, on the whole, a very good one, and while it will instruct the inexperienced surgeon, it contains many hints for the experienced one.

*Rete Mirabile in the Sloth.* From the article ANASTOMOSIS by Dr. Wardrop, we may extract the following observations on the *arterial rete* of the sloth. He will be seen to advance a different hypothesis from that generally received, with respect to the objects attained by it.

"While these forms of anastomosis are had recourse to, in the examples which I have given, in order to prevent an undue quantity of blood being sent to particular organs, I have also mentioned that a similar form of structure is found to be employed for the purpose of securing a proper quantity of blood to a part which, from the habits of some animals, would be otherwise occasionally deprived of its requisite quantity of the sanguineous fluid. The most remarkable illustration of this provision is met with in the limbs of the Sloth, the arteries both of the legs and arms of these animals being formed into a *rete*—to which, however, Sir Anthony Carlisle, who first described this structure, seems to me to have given a very erroneous and unphilosophical explanation, attributing to this mode of distribution in the arteries, the remarkably slow movements of this tribe of animals. The singular and no less curious part of the natural history of the sloth, is not, however, its extremely sluggish motions, which Sir A. Carlisle conceived could be explained from the peculiarity in the distribution of the brachial and femoral arteries, but the great muscularity which it possesses in grasping, or of suspending its own body for a most extraordinary length of time. 'We are told of a sloth that, having fastened itself by its feet to a pole, remained in the situation during fifteen days, and without sustenance! The strength in the legs and feet is so great, that having seized anything, it is almost impossible to oblige this animal to quit its hold. The same sloth grasped a dog that had been let loose upon it, and held it until it perished with hunger!'

The multiplied subdivisions of the brachial and femoral arteries of the sloth, and the subsequent reunion of these vessels into one trunk, must, therefore, be considered as a peculiarity of structure connected with the extraordinary power which these animals possess of clinging to branches of trees for a very prolonged time, and which they are impelled to do, in order to gather their food. Did the artery in the limb consist of one trunk, and was it in the usual situation, it is evident that the circulation would be disturbed, if not altogether interrupted, when the animal grasped with its limbs for any lengthened period; but all such compression is completely averted by the subdivisions of the brachial artery being so numerous at the part of the limb where the pressure is made, that whenever any of the vessels are compressed, and the circulation through them impeded, the blood finds a ready channel in the other branches of the trunk. There is even a further provision to ward off the effects of pressure on the brachial arteries of this animal, and which is so well known in the *feline* tribe,—the artery passing through a foramen on the inner condyle of the humerus; which may, indeed, be considered as an additional proof of the office which is required of the arterial plexus of the artery in the limbs."

Before we quit this Cyclopædia, we must recommend it to our readers. It is got up with much care on the part of its editor, and deserves encouragement. The greater *that* is, the better *it* will be.

### II. THE CYCLOPÆDIA OF ANATOMY AND PHYSIOLOGY. Edited by *R. B. Todd,* M. D. &c. &c. Part XI. August, 1837.

THIS work maintains its character, no trivial praise. The present number is almost exclusively devoted to human anatomy, and embraces the following subjects:—Eye (concluded), by Dr. Jacob—Face, by Mr. Partridge—Fascia, by Dr. Todd—Fat, by Mr. Brande—Femoral Artery, by Dr. Alcock—Fibrine, by Mr. Brande—Fibro-cartilage, by Dr. Todd—Fibrous Tissue, by Mr. Grainger—Fibular Artery, by Dr. Todd—Fifth Pair of Nerves, by Dr. Alcock.

These subjects are more or less ably handled by the gentlemen enumerated, but the details are in general far too rigidly anatomical for our pages. All that we can do is to select one or two statements, which either convey new facts or offer novel opinions.

1. *Size of the Posterior Chamber of the Aqueous Humor, and Operation of breaking up Cataract.*

The following observations on this subject are by Dr. Jacob. They deserve the attention of oculists.

" The size of the posterior chamber has been the subject of much discussion and controversy, and various attempts have been made by freezing the eye and other means to determine the matter. Petit, after a careful investigation, considered that the distance between the lens and iris was less than a quarter or half a line, in which Haller appears to concur. Winslow, in the Memoirs of the French Academy for 1721, insists that the iris is in contact with the lens. Lieutaud, in his *Essais Anatomiques,* is equally positive on this point, and even denies altogether the existence of a posterior chamber. The question is not an indifferent one, inasmuch as it involves important considerations as to operations for cataract and inflammations of the iris. Modern anatomists appear, generally, to consider the distance between the lens and iris to be greater than it really is. Although I cannot agree with Winslow and Lieutaud that the margin of the pupil is always in contact with the lens, I believe it frequently is so, especially in the earlier periods of life, when the curvatures of the lens are considerable. In iritis adhesions generally take place between the margin of the pupil and the capsule of the lens, a consequence not easily accounted for, if the parts be not in contact. In old age the lens becomes much flattened, and therefore retreats from the pupil, to such a degree that the shadow of the iris may often be seen in a crescentic form on a cataract; and in such persons, whether from this cause or from the inflammation not being of the adhesive character, blindness is more frequently attended with dilated pupil. In breaking up cataracts through the cornea, I have repeatedly satisfied myself of the contact or close vicinity of the two surfaces by placing the needle between them."

A sketch copied from the works of Sömmerring shews how small he considered the space between the iris and lens, and displays accurately how the posterior chamber is formed by the iris anteriorly, the lens posteriorly, and the ciliary processes at the circumference, with the small circular portion of the hyaloid membrane of the vitreous humour between the ciliary processes of the choroid and the circumference of the lens.

"It appears to me unaccountable," continues Dr. Jacob, "why surgeons, with these anatomical facts before them, still continue to introduce the needle into the posterior chamber, to break up cataracts, instead of passing it through the cornea into the anterior chamber, where ample space exists, and a full view is obtained of all the steps of the operation. In doing so the needle is thrust through opaque parts among delicate structures, into a narrow cavity, where, hidden by the iris, it can be used with little certainty of correct application. At the same time, instead of penetrating the simple structure of the cornea, which bears injury as well as any other structure of the body, the instrument pervades the fibrous sclerotic, a structure impatient of injury and prone to inflammation, punctures the ciliary ligament at the imminent risk of injuring one of the ciliary nerves or even wounding the long ciliary artery, and finally passes through one of the most vascular parts in the body, the *corpus ciliare*. The practice appears a signal instance of the influence of education, habit, and authority in setting improvement at defiance. The proofs afforded of the close vicinity of the margin of the pupil to the capsule of the lens, should remind the surgeon that one of the greatest dangers to be apprehended in iritis is the adhesion of these two parts, and that one of the first steps in the treatment should be to separate them by the application of belladonna, which, by its peculiar influence on the pupil, dilates that aperture, and, consequently, brings its margin more opposite the circumference of the lens and at a greater distance from the prominent central portion."

2. *Comparative Anatomy of the Two Portions of the Fifth Pair of Nerves.* Dr. Alcock, the author of the article on the Anatomy of the Fifth Pair of Nerves, thus alludes to the comparative anatomy of its ganglionic or sensitive and non-ganglionic or motor portions.

"The distinction of the nerve into two portions appears to prevail uniformly throughout the animal series. According to M. Serres, it is to be observed in all the classes of the vertebrate animals except the Reptiles; but in them, according to him, the *lateral fasciculi* are wanting. The latter assertion, however, is incorrect, the distinction being to be observed as satisfactorily in that class as in any other. Again, the distinction is not equally remarkable in all; in some it is still more so than in man; in others it is less; and according to the same authority, it is to be observed among Mammalia the more easily as we pass from Man to the Rodentia. Among the Cetacea it is divided throughout into two separate fasciculi."

3. *True Origin of the Motor Portion of the Fifth Nerve.* "In none of the authorities which the author has had an opportunity of consulting, has he found a particular origin assigned to the lesser packet. By most anatomical writers it is overlooked; J. F. Meckel states that it can be traced a certain way into the crus, but he goes no further; Mayo asserts that the lesser portion arises close upon the greater, and, in a sketch of the origins of the nerves given by him in his Physiology, it is represented traversing the crus cerebelli, as a single fasciculus, above and behind the greater, and attached to some part above that from which the greater is represented to arise: but still the origin is not defined, and it is manifestly intended to be distinct from that of the greater packet.

The author has succeeded, as it appears to him satisfactorily, in tracing both the roots of the lesser packet to a destination for which he was not prepared; at setting out he expected to have found the origin of the lesser different from that of the greater packet, and to have followed it to a prolongation of the anterior columns of the spinal cord, as has been stated by Harrison; it was therefore with surprise that, after a patient dissection, he succeeded in tracing both its roots to the same point, to which the greater packet is attached, behind the middle crus of the cerebellum; both the roots traverse the crus, as the greater does, the inferior very frequently in company with and internal to the greater packet, or separated from it by a very thin stratum of the substance of the crus, the superior near to the superior surface of that part, and separated from the greater packet by an interposed stratum of two or more lines; the course of the latter is so near to the surface of the crus, that it can frequently be traced for a considerable way by the eye without dissection: they present, in their mode of traversing the crus, two remarkable varieties; in some instances the fasciculi, of which they are composed, are separated from each other and even interlaced with those of the crus, and in such the pursuit of them is intricate and difficult; in others they pass in two distinct packets, and in these they are more easily followed. As they proceed they approach the greater packet, so that the interval between them and it gradually diminishes, and having traversed the crus, they are both attached below and behind it to the same part as the greater packet, and posterior to it. This view of the connection of the lesser packet, if confirmed, must lead to interesting results with regard to the relations of the two portions of the fifth nerve at least; it will at all events decide the question as yet in dispute, whether they are to be regarded as distinct nerves, or parts of the same; upon this point further light will be thrown by the disposition of the same part in fish, in which the source of the uncertainty prevailing with regard to the nerve in the higher classes does not exist to the same amount; inasmuch as the ganglionic and non-ganglionic divisions of the nerve seem for the greater part associated in their distribution."

Whether this is correct or not, remains to be established by other anatomists. The preceding are the principal remarks of novelty in this number of the Cyclopædia.

---

A NEW MODE OF TREATMENT EMPLOYED IN THE CURE OF VARIOUS FORMS OF ULCER AND GRANULATING WOUNDS. By *Frederick C. Skey*, F.R.S. &c. &c. 8vo. Stitched, pp. 85.

ALL our readers know what numerous brochures have appeared, are appearing, and probably will still continue to appear, on the subject of the treatment of ulcers. Mr. Skey's present pamphlet adverts to the number of its predecessors, and while it does not deprecate their value, it hopes to add something to the stock of remedies they have proposed. Indeed it does something more, for Mr. Skey observes in his Preface:—

"It is more that I fear the views I advocate may be prejudiced by an apparently overweening confidence on my part, than from any distrust of the future, confirming the confidence I entertain in the proposed remedy, that I hesitate to avow my unqualified belief in its competence to invariably supersede *all* operations, in the treatment and removal of non-specific ulcers.

With a careful diagnosis, a discriminating and judicious employment of the means, I believe, henceforth, the knife will be wholly unjustifiable."

After passing in review the several kinds of ulcers which occur, Mr. Skey particularizes that to which his mode of treatment is peculiarly applicable:—

"Although I conceive that the treatment I shall afterwards recommend, will be found applicable to every form of ulcer dependent on weakness—and in this catalogue I should include more than nine-tenths of those affecting the lower limbs, and a large proportion of ulcers situated on any other part of the body —yet there is none in which it will be found so striking as in that most frequent form of the chronic or callous ulcer, affecting the legs of old persons.

The true chronic ulcer is destitute of all action of a healthy kind. It may co-exist with varicose veins, or it may not."

But what is the plan? It is the exhibition of opium. Mr. Skey quotes a passage from the "Opium eater," to shew its physiological effects on the morale, and then adds another of his own, establishing the parallel in regard to the physique. We will not absolutely guarantee the physiology of either the one or the other, but our readers shall judge for themselves.

" It communicates," says M De Quincy, " serenity and equipoise to all the faculties, active and passive; and with respect to the temper and moral feelings in general, it gives simply that sort of vital warmth which is approved by the judgment." " The expansion of the benign feelings incident to opium *is no febrile access, but a healthy restoration to that state which the mind would naturally recover upon the removal of any deep-seated irritation of pain* which had disturbed and quarrelled with the impulses of a heart originally just and good."

In the same degree that opium soothes and tranquillises the moral, does it invigorate the physical frame, and animate the dormant energies of healthy action. While its beneficial influence on the one, demands the existence of some mental obliquity, some deviation from the standard of health, so the other, requires the previous existence of actual disease, the ravages of which demand assistance, from the powers of renovation."

It is in small doses that it produces this desirable effect, and, in small doses, Mr. Skey prescribes it—half a grain, for instance, night and morning, in open and discharging wounds.

" In many cases a very palpable effect is produced by eight drops of the tincture, twice a day; but I rarely commence with a less dose, than half a grain of the extract night and morning. The quantity will, however, depend on the age, sex, and constitution of the patient. Old persons bear opium better than young, and occasionally persons exhibit a peculiar idiosyncrasy, precluding its use altogether. An old person, varying from fifty to upwards, whose pulse is languid, whose skin is cool, and whose nervous system has been debilitated by indulgence in spirituous liquors, will take opium to almost any *medical* amount, not only with impunity, but with striking advantage. Yet in every case, however favourable in appearance, I commence with the dose above mentioned, viz. half a grain to two-thirds. On these conditions, however, it may be rapidly increased, if necessary, up to two grains night and morning."

" I do not," says Mr. Skey, " profess to cure every, nor indeed any, description of ulcer by means of opium. There is great difference between *curing* and *healing* an ulcer. The term ' to cure' is much more comprehensive in its application, and embraces every variety of change, whether healthy or morbid, through which an ulcer travels to the final process of perfect cicatrisation. The term to heal is applicable only to the last stage, including the secretion of lymph, its organisation by vessels and nerves, and the investing process by the formation of skin; and this power I claim for opium under certain essential restrictions."

Most surgeons, we conceive, are in the habit of employing opium for certain ulcers, for those attended with much pain, or inclined to assume phagedænic characters, or occurring in the old and the debauched. So far then Mr. Skey's recommendation of the medicine cannot claim the merit of much originality. But he certainly appears to use it much more extensively than this, and there are not in his pages any material restrictions to its use in open and discharging sores.

Such treatment, we fancy, will be found more beneficial and less hazardous in the class of persons who enter hospitals for bad legs, than in the cases of individuals in the better classes of society. In them the great requisite is to improve the secretions, and gently to stimulate them while we do so. We doubt, therefore, whether Mr. Skey's plan will be found so universally applicable as he anticipates.

---

### NATURAL HISTORY.

1. A HISTORY OF BRITISH QUADRUPEDS. By *Thomas Bell*, F.R.S, F.L.S. Professor of Zoology in King's College, London. Illustrated by a Wood-cut of each Species, and numerous vignettes. Published in Parts, price 2s. 6d. each. Parts 1 to 10.

2. A HISTORY OF BRITISH BIRDS. By *William Yarrell*, F.L.S. Secretary to the Zoological Society. Illustrated by a Wood-cut of each Species, and numerous vignettes. 8vo. Stitched. Van Voorst, London. July, 1837. Part I. Price 2s. 6d.

ONE number more, and the first work on our list will be complete. That number was to have been published on the 1st of August, but we have not yet, and it is the latter end of the month, received it.

The work itself is a highly interesting, we may say a valuable one. The execution is in every respect creditable to *all* the parties concerned—author—engraver—printer—and publisher. We strongly recommend such of our readers as take an interest in natural history, and particularly our readers in the country, to purchase the volume when completed. It will be equally elegant and entertaining in the professional library or on the table of the drawing-room.

The first Part of a History of British Birds by Mr. Yarrell is a fit companion to the work of Mr. Bell. Mr. Yarrell's situation as Secretary to the Zoological Society gives him necessarily considerable opportunities of which he knows well how to avail himself.

In this Part we are presented with an interesting account, and spirited wood-cuts of the Egyptian vulture—the golden eagle—the white-tailed eagle—the osprey—the gyr falcon—the peregrine falcon—the hobby—and the red-footed falcon.

As a sample of Mr. Yarrell's mode of treating his subject take the following passages.

" The power of vision in birds is observed to be very extraordinary; and in none is it more conspicuous than in the Eagles, and in the *Falconidæ* generally. It has been stated that probably in the whole range of anatomical structure, no more perfect or more conclusive proofs of design could be adduced than are to be found in the numerous and beautiful modifications in the form of various parts of the eyes of different animals, destined to exercise vision in media of various degrees of transparency as well as density. The figure on the right hand of the vignette at the end of this article represents the circle, composed of fifteen bony plates, by which the orb of the eye of the Golden Eagle is supported. These bony plates are capable of slight motion upon each other. The figure on the left hand in the vignette represents the crystalline lens of the same bird; the lens being subject to great variety of form in different birds. In the Eagle, the proportion of the axis to the diameter of the lens is as three and eight-tenths to five and seven-tenths; in the Great Owl, which seeks its prey at twilight, the relative proportions of the lens are as six and five-tenths to seven and eight-tenths; and in the Swan, which has to select its food under water, the proportions of the lens are as three to three and eight-tenths. Birds have also the power of altering the degree of the convexity of the cornea. With numerous modifications of form, aided by delicate muscular arrangement, birds appear to have the power of obtaining such variable degrees of extent or intensity of vision as are most in accordance with their peculiar habits and necessities."

"'The versatility of the outer toe of the Osprey, the strength, curvature, and sharpness of its claws, and the roughness of the soles of its feet, are peculiarities of structure adapted to the better securing its slippery prey; and the shortness of its thigh-feathers, unusual in the Falcon tribe, is also evidently connected with its fishing habits.' A specimen at the Gardens of the Zoological Society of London, when a fish was given to it, was observed to seize it across the body, placing the inner and outer toes at right angles with the middle and hind toes, and digging in the claws, held the fish most firmly by four opposite points; not relaxing its hold or altering the position of the toes, but picking out the portions of flesh from between them with great ease and dexterity."

We recommend this work too, and shall notice successive parts, as they appear.

---

On the Nature and Treatment of the Diseases of the Heart, with some new Views on the Physiology of the Circulation. By *James Wardrop*, M.D. Part the First. Churchill, 1837.

The Author commences this part with some description of the anatomy of the heart; but commits a physiological exaggeration in the very beginning.

"There is, however, one essential difference in the functions of the muscles of the heart from those of all other organs; they *have no repose!*"

There can be no question that the contractions of the heart's muscular fibres are periodical. The systole requires about one half the space of time required by the diastole—hence the act of *contraction* occupies about eight out of the twenty-four hours. Is this constant action? We agree with Dr. Wardrop that the apparent effect of muscular exercise is to accelerate the venous, and somewhat retard the arterial circulation.

"But whilst the pressure caused by muscles during their contraction propels the blood onwards in the contiguous *veins*, it seems never to have been contemplated what must be the effect of that compression on the adjacent *arteries*, although these vessels are doubtless alike exposed to its influence. It will however be shown that the effect of muscular contractions both on arteries adjacent to, as well as on those imbedded in, the substance of muscles, must be to compress these vessels, by which compression the flow of blood through them will be necessarily impeded,—hence, the contraction of muscles will increase the accumulation of blood within the heart in two ways,—by *accelerating* the flow of the venous blood to the right heart, and by *impeding* the transit of the arterial blood from the left heart."

It would not be difficult, we conceive, to prove, that, owing to the powerful *vis à tergo* propelling the blood in the arteries—the elastic nature of their coats—and the disposition of the valves in the veins, the compression exerted by the muscles cannot tell greatly against the circulation.

But Dr. Wardrop carries out his ideas on this subject rather far. Few would be inclined to imagine that this pressure of the muscles on the arteries does great things in the economy. Yet Dr. Wardrop assures us that it does so.

"For performing this important function, and which I have denominated the *musculo-cardiac function*, we perceive several simple and beautiful contrivances; whilst we also find a striking illustration of this peculiar office of the muscular system in the mechanism which nature has adopted in order to *prevent* the compression of some arteries, when such compression would be injurious to the proper performance of the functions of those organs which such arteries supply. For although it appears that it is not requisite that some organs should be at all times supplied with an equal quantity of blood, there are others wherein any alteration in the supply of blood, would be prejudicial, or even fatal, to the great functions of life; and hence, whenever the heart requires an additional quantity of blood, that office is fulfilled by impeding the flow of the arterial blood through the arteries of those organs only which do not at all times require a uniform supply of the sanguineous fluid.

In accordance with these positions, we find that the arteries of all organs of the first denominations are so placed that they must inevitably be more or less compressed by the contractions of the adjacent muscles; whilst the arteries of the other class of organs are so situated, that they are protected from all pressure from the movements of the muscles contiguous to them.

For a demonstration of the first of these conditions, we must look at the arteries of the limbs, while those of the brain, heart, stomach, and iris, each exemplify particular contrivances, by means of which the quantity of blood in these organs is not subject to any variations from muscular movements.

There are, indeed, several points, in the anatomy both of the muscles and of the arteries, which seem to be specially subservient to the musculo-cardiac function. Arteries accompany the veins where it is intended that *both* these systems of vessels shall be influenced by muscular contractions; yet, long as the period has been that the relative position of the blood-vessels and muscles of the limbs has been pointed out, no rational explanation of the utility of such an arrangement has been given. It is, however, evident, according to the preceding views, that, by such a disposition of the two systems of vessels, the velocity of the blood is equally influenced in the veins and in the arteries whenever the adjacent muscles are contracted;—that whilst muscular contractions assist in propelling the blood in the veins forwards to the right heart, the valves preventing its regurgitation, the same pressure impedes the current in the contiguous arteries, and thus diminishes the exit of blood from the left heart.

Here, then, we have an anatomical fact, affording additional proof of the simplicity and wisdom which Nature displays in all her works, employing one organ to perform at the same time more than one function,—veins and arteries accompanying each other in those situations where it is intended that the circulation of the blood, both venous and arterial, shall be influenced by the contractions of muscles; at the same time those vessels which are *not* liable to compression from muscular contractions are not similarly disposed, either with relation to the muscles or to each other;—thus in the internal viscera, such as in the brain, lungs, and liver, the veins do not accompany the arteries."

This is ingenious, but, as our trans-Atlantic contemporaries say, it wants confirmation. Nay, the facts which are set forth are dubious. Dr. Wardrop, for example, observes, that, in the limbs, there is no provision for obviating the pressure of the muscles on the arteries. Suppose we take the main arteries of the lower limbs, and observe whether this statement, in reference to them, is accurate. The inguinal artery passes through a tendinous canal at the groin; it does the same where it passes to the back of the thigh; it lies deep in a bony gutter at the ham; it passes under a tendinous arch beneath the soleus; the profound and perforating branches all go through tendinous openings in the adductor muscles. Do these demonstrable contrivances square with our author's observations, or do they look as if Nature rather wished for pressure on the vessels than otherwise? They rather tend, to a plain thinker, to shew that where any amount of compression might be anticipated, means are employed to obviate it.

It must be remarked, too, that in all these instances the artery is accompanied by its vein, a circumstance which also militates against the spirit of Dr. Wardrop's remarks. No doubt that the muscular compression *does* impede, in some degree, the passage of the blood through the arteries. But we may grant this, without admitting that either the degree of that impediment is so great as Dr. Wardrop appears to suppose, or that it serves so useful a purpose as he contends for in the system. To us it appears rather to be a necessary consequence of the existence of arteries and muscles, which Nature has rather guarded against than favoured by specific arrangements, and which she rather submits to, than turns to any particular purpose. But our author's words have been quoted, and our readers may possibly prefer his ideas to the prevailing ones.

Dr. Wardrop applies his theory to the explanation of many important phenomena, which, without its assistance, were, he thinks, inexplicable.

"Some muscular movement will be observed to precede every effort which we make to increase the action of particular organs, because, for such a purpose, it is first required that the vigour of the heart shall be increased. Hence we find that, unless when the body is in a state of perfect tranquillity, which can scarcely ever happen but during sleep, the circulation of the blood throughout the system is constantly varying, any muscular movement, however slight, increasing the influx of the venous blood, and at the same time impeding the reflux of the arterial blood. Thus in some organs the quantity of blood is diminished, and in others increased, by every motion of the body, those which are called upon to perform particular functions receiving an increase, whilst other organs whose office is less urgent, experience a diminution in the supply of the sanguineous fluid."

"Observe how the strength and vigour of the body is increased when we require to make any great exertion! In order to produce a temporary increase in the heart's action, I have already remarked that it is requisite to give to that organ an additional stimulus. This is at once effected by throwing into action such muscles as shall, by their newly acquired forms, compress the contiguous arteries, and thus impede the transit of the arterial blood.

If, by way of illustration, a man be about to make any great exertion, such as running or leaping, he prepares himself, as it were, by first vigorously contracting the muscles of the arms and clenching his hands. For the same reason, when a person is subjected to pain, as that of a surgical operation, he prepares himself to endure it by throwing into action almost all the voluntary muscles, grasping firmly with his hands, and pressing the feet against some resisting body. And when the female, during parturition, is about to make a powerful expulsive effort to assist the uterus in giving birth to the infant, she in like manner throws into violent and long continued contractions the muscles of the extremities, clenches the jaws, and squeezes with a convulsive effort whatever may be within her reach."

Dr. Wardrop takes no notice of an obvious method of accounting for the circumstances that he mentions. If a person is about to make a violent or continued effort with some muscles, it is necessary that their points of origin should be firmly fixed, and that the fulcra of the bony levers to be moved should be fixed too. Other muscles are called into action to effect this, and the very instance cited by our author—that of the female in the act of parturition—is precisely one in point. We fancy that this mechanical object is one of, at least, as much importance, as that which Dr. Wardrop dwells on.

However this may be, our author applies his physiological views to pathology. By the former, we are shewn that yawning and stretching are very important natural means of *waking* us, that is, of restoring energy to the sleeping sensorium—by the latter, that the same yawning and stretching are not always to be lightly indulged in.

"My attention was directed to some of these phenomena and to the physical explanation of them which I have now given, when attending a patient who had frequent attacks of gout, and who had been sometime before affected with an arthritic inflammation of the pericardium. After that illness he suffered from palpitation of the heart every morning just at the moment when he awoke, but they continued only a few minutes. On enquiring if he was in the habit of yawning and stretching his limbs, he then stated that he always did so whilst in the act of awaking, and that it was immediately afterwards when he felt the palpitation. He further observed, when interrogated on this point, that, if he accidentally awoke during the night and stretched himself, palpitation of the heart always ensued.

The effect of yawning and stretching the body in disturbing a diseased heart I also observed in a person who had for many years suffered from symptoms of Hypertrophy, and who was recovering from a febrile illness, for the relief of which an antiphlogistic treatment had been pursued. When convalescent, this patient had no uneasy feelings remaining except a pain in his head, which came on every morning immediately after he awoke, but did not continue longer than ten minutes. From the circumstance of this head-ache occurring only after he awoke, and from his stating that he was in the habit of yawning and forcibly stretching his limbs whilst he was awaking, it appeared to me probable that the increase of blood in the irritable heart caused by those movements, produced the *headache* in the same manner as it had in the former instance caused *palpitation*."

Dr. Wardrop passes to the lungs, and, after noticing the effects of inspiration and expiration on the circulation, he proceeds to dwell on what he terms the *pulmo-cardiac function.* This essentially consists in the capability on the part of the lungs, of receiving a surplus quantity of blood which would otherwise injuriously distend the heart.

Dr. Wardrop goes on to observe that whilst the Pulmo-Cardiac Function is employed to relieve the heart of any surplus quantity of blood which it cannot receive within its cavities, the *veins* will in like manner be found to relieve the pulmonary vessels of any superabundant blood which they are not capable of receiving without interruption to the great function of respiration.

The veins which effect this purpose are the subcutaneous veins, those which are not compressed by the muscles in their contraction. No more striking example, he observes, can be given of this office of the superficial veins, than their distended appearance beneath the delicate skin of a race-horse, after a severe gallop.

Dr. Wardrop considers seriatim the process of training—the influence of laughing—crying—weeping—sobbing—sighing—vomiting—coughing—sneezing—and hiccup, in effecting certain changes in the circulation of the blood in the lungs and heart for some essential purpose. He treats also of the modifications of respiration in running and in raising a weight, and he thus obscurely explains sea-sickness :

"It is from a want of the power of adjustment, or co-operation in the respiratory and circulating organs, by which we can explain *sea-sickness*. Dr. Wollaston observed, that in those who did not suffer sickness at sea, their respiration was altered. In waking from very disturbed sleep, he found that his respirations were not made with the accustomed uniformity, but were interrupted by irregular pauses, with an appearance of watching for some favourable opportunity to make the succeeding effort, and it seemed as if the act of inspiration were in some manner to be guided by the tendency of the vessel to pitch with an uneasy motion."

He terminates the consideration of the Pulmo-Cardiac Function, by a few remarks on the effects of swinging on the circulation, on the preservation of the balance of the circulation in different postures, and on the modification of the circulation according to the habits of different animals.

The work concludes with some general reflections on the reciprocal influence of the heart and brain ; on the reciprocal influence of the heart and digestive organs ; on the blood ; on the pulse ; and on the sounds of the heart. None appear to require notice, and we quit the work, with a recommendation to such of our readers as desire to peruse some very ingenious speculations on important phenomena, to purchase and to study it. The observations it contains are couched in an easy unaffected style, and many are extremely interesting.

